# The Book World of Medicine and Science

**Published:** 1896-09-12

**Authors:** 


### THE BOOK WORLD OF MEDICINE AND SCIENCE.

A Text-book of Histology: Descriptive and Practical. By Arthur Clarkson, M.B., C.M.Edin., with 174 Original Coloured Illustrations. Bristol: John Wright and Co. 1896. Price 21s. net.

The author of this book has set himself a task which is both interesting and praiseworthy. His aim has been to combine in one volume both the descriptive and the practical parts of the science of histology, and we are sure that anyone who will carefully read this work and study the admirable drawings by which it is illustrated will agree with us in saying that he has well fulfilled the task he set himself.

It is easy to say that hundreds of people wish to know the minute structure of the animal frame who can never have any necessity, or in fact any opportunity, of practising the methods by which the microscopic anatomy of the body has been worked out. But there is this difference between mere anatomy and histology. Anatomy describes what is visible to the eye in the dissected body, but the histologist would have but a small tale to tell did he not prepare and stain the tissues by processes which are often somewhat complex and elaborate, and in no small number of cases the interpretation of what one sees depends largely on one's knowledge of the processes to which the specimen has been subjected. Every year what one may call picture-book histology becomes more and more artificial, and we think the author has done well to combine in one work both the results arrived at and the processes by which they have been reached, by a knowledge of which alone they can be properly interpreted.

In the first two chapters the general methods of histological examination are given, after which in each chapter the structure of the tissue or organ dealt with is first systematically described, then the specimens illustrating it are demonstrated, and, finally, a description is given of the methods of preparation. The teaching of the author is curiously, one might fairly say interestingly, tutorial. The desire to explain is evidently strong within him, and he does not hesitate to speak of function when it seems explanatory of the subject more especially in hand.

The most interesting and special feature in the book, however, lies in the demonstrations of the structure exhibited in the specimens. The illustrations are remarkably good. The fullest use has been made of chromo-lithography, with the result of placing before the student a series of striking reproductions of microscopic sections of the various tissues and organs. Some, even of the coloured illustrations, are mere diagrams or are "semi-diagramatic," but the mass of them are obviously drawings from nature of the specimens described, and when the student has been "taken tutorially over the specimens," as he is in each chapter, he obtains a much more accurate familiarity both with the structures and with the appearances they present under the microscope—two very different things—than he would be likely to derive from a study of mere text or of less perfect illustrations.

It is hardly necessary to say that there are many points in histology which are matters of dispute, and it is not to be expected that any writer will in all points meet with universal acceptance, but we think that Dr. Clarkson has produced an orthodox book on which students may safely rely, although, perhaps, here and there at the expense of omitting controversial points which would be of interest to more advanced histologists. It is a book on which we may fairly congratulate both the author and the publishers.

